# Actual Racial/Ethnic Disparities in COVID-19 Mortality for the Non-Hispanic Black Compared to Non-Hispanic White Population in 35 US States and Their Association with Structural Racism

**DOI:** 10.1007/s40615-021-01028-1

**Published:** 2021-04-27

**Authors:** Michael Siegel, Isabella Critchfield-Jain, Matthew Boykin, Alicia Owens

**Affiliations:** Department of Community Health Sciences, Boston University School of Public Health, 801 Massachusetts Avenue, Boston, MA 02118 USA

**Keywords:** COVID-19 (coronavirus disease 2019), Health disparities, Structural racism, African Americans, Age-adjusted mortality rates

## Abstract

**Introduction:**

While the increased burden of COVID-19 among the Black population has been recognized, most attempts to quantify the extent of this racial disparity have not taken the age distribution of the population into account. In this paper, we determine the Black–White disparity in COVID-19 mortality rates across 35 states using direct age standardization. We then explore the relationship between structural racism and differences in the magnitude of this disparity across states.

**Methods:**

Using data from the Centers for Disease Control and Prevention, we calculated both crude and age-adjusted COVID-19 mortality rates for the non-Hispanic White and non-Hispanic Black populations in each state. We explored the relationship between a state-level structural racism index and the observed differences in the racial disparities in COVID-19 mortality across states. We explored the potential mediating effects of disparities in exposure based on occupation, underlying medical conditions, and health care access.

**Results:**

Relying upon crude death rate ratios resulted in a substantial underestimation of the true magnitude of the Black–White disparity in COVID-19 mortality rates. The structural racism index was a robust predictor of the observed racial disparities. Each standard deviation increase in the racism index was associated with an increase of 0.26 in the ratio of COVID-19 mortality rates among the Black compared to the White population.

**Conclusions:**

Structural racism should be considered a root cause of the Black–White disparity in COVID-19 mortality. Dismantling the long-standing systems of racial oppression is critical to adequately address both the downstream and upstream causes of racial inequities in the disease burden of COVID-19.

**Supplementary Information:**

The online version contains supplementary material available at 10.1007/s40615-021-01028-1.

## Introduction

No country in the world has suffered a greater burden from COVID-19 mortality than the USA, which has experienced 534,000 deaths as of the middle of March 2021, accounting for 19.6% of the worldwide total [1]. Within the USA, the burden of COVID-19 death has not been experienced equally by all racial/ethnic groups. People who are Black are dying from COVID-19 at 3.6 times the rate of people who are White, and people who are Hispanic, non-Hispanic American Indian/Alaska Native, and non-Hispanic Asian/Pacific Islander are also experiencing disproportionately high COVID-19 mortality rates (with death rate ratios compared to the White population of 2.8, 2.2, and 1.6, respectively [2]. While racial disparities in health outcomes have always existed, COVID-19 has brought the starkness of these disparities to the public eye in a uniquely visible way [3]. In turn, the pandemic has focused attention on the potential role of structural racism in creating and exacerbating racial disparities in health. Although many studies have investigated the overall national racial disparity in COVID-19 mortality, few have explicitly compared race-specific mortality rates within states. Similarly, although many studies have suggested a role for structural racism in explaining these disparities, few have empirically demonstrated such a connection by attempting to actually quantify structural racism as a specific measure.

Most existing studies that have demonstrated racial disparities in COVID-19 mortality have done so in one of three ways: (1) showing that the number of people in specific racial/ethnic groups dying from COVID-19 exceeds their representation in the overall population [4, 5]; (2) demonstrating that the percentage of a non-White racial/ethnic group in a county, city, or neighborhood significantly predicts the overall COVID-19 death rate in that county, city, or neighborhood [6–24]; and (3) quantifying death rates among individual patients of different racial/ethnic groups in clinical settings such as hospitals, health systems, or regional or national registries [25–29]. Surprisingly, only a limited number of studies have explored racial disparities in COVID-19 mortality by explicitly calculating and comparing death rates across racial/ethnic groups. Of these, only a few considered the age distribution of the population by generating age-specific mortality rates.

Age is perhaps the single most important predictor of COVID-19 mortality [30]. Because different racial/ethnic groups have different population age distributions, comparing crude COVID-19 death rates can be dangerously misleading. For example, in Minnesota, people who are White account for 80% of the population and 82% of COVID-19 deaths, suggesting that there is no racial disparity [30]. However, after adjusting for age, the Black population was found to have COVID-19 death rates that were 5.3 times higher than the White population [30]. The misleading impression from the crude death rate comparison could be dangerous because it may lead to the conclusion that there is no need to address racial disparities and therefore no need to examine deeply underlying structural conditions that have made one racial group more than five times more likely to die from this virus. Similarly, another study found that among four academic hospitals in Atlanta, the odds of Black patients dying from COVID-19 were actually lower than those of non-Black patients (the death rate ratio was 0.9) [31]. However, after adjusting for age, Black patients were 1.5 times more likely to die. Because the Black population tends to be younger than the White population, unadjusted COVID-19 death rate disparities are underestimates of the true disparities [32, 33]. The American Public Media (APM) Research Lab has demonstrated that while the ratio of crude national COVID-19 death rates among Black people compared to White people is just 1.4, the ratio is 2.3 after indirect age standardization [33].

Perhaps the most widely used tool to understand racial disparities in COVID-19 mortality is the COVID-19 Racial Data Tracker [34]. While this is an immensely useful tool, it is limited because it does not present age-adjusted death rates. Thus, the racial disparities presented by the tracker are greatly underestimated. For example, the Racial Data Tracker reports that Black people in Minnesota make up 6% of the population but account for only 5% of COVID-19 deaths, making it appear that there is no racial disparity [34]. However, as shown earlier, after accounting for age, the Black population in Minnesota is actually dying at a rate that is more than five times higher than that of the White population [30]. This demonstrates the critical need for studies that compute and compare age-adjusted COVID-19 death rates across racial groups.

Several previous studies have examined racial disparities in COVID-19 mortality using crude death rates [35–38]. Two studies reported national, race-specific COVID-19 mortality rates without age adjustment [35, 36]. Two presented state-specific estimates of the crude COVID-19 mortality rate by racial/ethnic group [37, 38]. We are aware of only three papers that have calculated age-adjusted, race/ethnicity-specific COVID-19 death rates [2, 39, 40]. Bassett et al. [2] reported that nationally, the age-adjusted COVID-19 death rate for the non-Hispanic Black population was 3.6 times higher than for the non-Hispanic White population. This study is limited, however, because it only presents national data. Goldstein and Atherwood [39] reported that on a national level, the Black population had an 80% higher age-adjusted COVID-19 mortality rate than the White population. Their estimate of the racial disparity is much lower than that of Bassett et al. because they used indirect rather than direct age standardization. This study also presents state-specific estimates of the racial disparity in COVID-19 mortality based on indirectly age-standardized death rates. Gross et al. [40] also present state-specific estimates of racial disparities in COVID-19 mortality rates using indirect age standardization. The APM Research Lab also provides state-specific estimates of racial disparities in COVID-19 death rates using indirectly standardized rates [33]. A summary of the previous studies that have presented state-specific estimates of the Black–White racial disparity in COVID-19 mortality rates is shown in Appendix Table 4.

The validity of indirect age standardization has been questioned because this method produces only an approximation of the age-specific mortality rates in a state and because it generates rate estimates that cannot necessarily be compared across different locations, such as states [33, 41]. The APM Research Lab points out that “Indirect standardization may deviate more from directly age-adjusted rates when comparing two populations that differ significantly in their age distribution, as race groups may. For this reason, data from individual states that are directly age-adjusted should be considered superior” [33]. Therefore, there is an urgent need for a state-level analysis of racial disparities in COVID-19 mortality based on directly age-adjusted death rates. The reason indirect standardization has been used in previous COVID-19 studies is that there has been a lack of state-level data on the age distribution of COVID-19 deaths by race/ethnicity. Recent improvements in reporting, however, make it possible to generate directly standardized rates for most states. This paper helps to fill this critical gap by reporting, for the first time, the magnitude of the Black–White racial disparity in COVID-19 mortality at the state level based on an analysis of age-adjusted death rates using direct age standardization.

With national age-adjusted death rates revealing an even more profound racial disparity in COVID-19 mortality, it is even more crucial to determine the reasons for these disparities. Although many papers have suggested that structural racism is a critical factor in explaining racial disparities in COVID-19 mortality, we are aware of only six that have demonstrated this relationship empirically by explicitly measuring structural racism [42–47]. Tan et al. [42] examined the relationship between four measures of structural racism (residential segregation and differences in incarceration rates, economic status, and employment status) and county-level COVID-19 death rates, finding that the degree of residential segregation was significantly related to higher overall death rates. However, a limitation of this study was that it did not actually calculate or model race-specific mortality rates. Five other studies examined the relationship between measures of structural racism and overall county-level COVID-19 mortality [43–47]. The measures used in these studies were the proportion of individuals living in “Black-concentrated” Census tracts [43], implicit and explicit racial attitudes [44], and racial residential segregation [45–47]. Like Tan et al. [42], none of these studies modeled race-specific mortality rates. This study helps to fill this critical gap by explicitly modeling the relationship between measures of structural racism and the Black–White racial disparity in COVID-19 mortality rates. The previous studies examined overall county- or tract-level COVID-19 mortality rates and estimated the effect of various population compositional characteristics, such as the percentage of racial/ethnic subpopulations. Directly modeling disparities in race-specific mortality rates is a preferred approach because the proportion of a certain racial/ethnic group living in a county could be related to any number of factors that might increase overall risk for COVID-19, including among White people. Thus, finding a relationship between, for example, the percentage of Black people in a county and the overall COVID-19 mortality rate does not necessarily demonstrate that there is a racial disparity. Here, we do not infer such a relationship but measure it directly.

This paper advances the previous literature in a third way. Calculating the Black–White disparity in age-standardized death rates among different states provides a standard unit of comparison across states, enabling a more pointed exploration of the reasons for these racial disparities. As Goldstein and Atherwood state: “state-level variation in disparities may help us to understand the causes of disparities in COVID-19 death rates. We find that the highest excess mortality for Blacks is the Deep South and the Upper Midwest, while disparities are smallest in the Northeast and West. Understanding the reasons for this pattern are an important topic for future research” [39 , p. 9]. This paper advances the literature by being the first to explicitly model the difference in the Black–White disparity in COVID-19 mortality across states, allowing us to investigate whether differences in the level of structural racism across these states help to explain the level of the disparity in health outcomes.

In this paper, we explore the relationship between structural racism and the Black–White disparity in directly age-adjusted COVID-19 mortality rates across 35 states. Understanding racial disparities at the state level is imperative because states have the primary responsibility for implementing policies related to the prevention, control, and response to COVID-19 and therefore are directly responsible for the emergence of, and amelioration of, racial disparities related to COVID-19 [48]. Identifying differences in the degree of the racial disparities across states may be enlightening because states have a very different history of their implementation of racist policies [49] that may help explain the cause of the observed racial disparities in COVID-19 mortality today. This paper responds to calls for the explicit investigation of the relationship between measures of structural racism and racial disparities in COVID-19 outcomes [50].

## Methods

### Design Overview

Using data from the Centers for Disease Control and Prevention’s (CDC) National Center for Health Statistics, we calculated both crude and age-adjusted COVID-19 mortality rates for the non-Hispanic White and non-Hispanic Black populations in each state. We defined the Black–White disparity in COVID-19 mortality as the ratio of the age-adjusted death rate among the Black population to the age-adjusted death rate among the White population. Using a previously developed state-level index of structural racism, we explored the relationship between this state racism index and the observed differences in the racial disparities in COVID-19 mortality across states by conducting linear regression analyses. Also, using linear regression analysis, we explored the potential mediating effects of three variables identified in the literature as possible explanations for observed racial disparities in COVID-19 mortality: disparities in exposure based on occupation; disparities in weathering effects based on the presence of underlying medical conditions that could increase COVID-19 severity; and disparities in health care access.

### Measures and Data Sources

#### Outcome Variable

The outcome variable was the Black–White racial disparity in age-adjusted COVID-19 death rates in each state, defined as the ratio of the non-Hispanic Black age-adjusted death rate to the non-Hispanic White age-adjusted death rate. In epidemiology terminology, this is known as the standardized rate ratio (SRR).

Data on confirmed COVID-19 deaths by age, race, and state were obtained from the National Center for Health Statistics’ COVID-19 Death Data and Resources [51]. We used the following data sets: (1) Provisional COVID-19 Deaths by Week (State); (2) Provisional COVID-19 Deaths by Sex, Age, and State; (3) Provisional COVID-19 Deaths by Race and Hispanic Origin (State); and (4) Provisional COVID-19 Deaths by Race and Hispanic Origin and Age (State). These data sets, updated weekly, represent provisional counts of COVID-19 deaths from the National Vital Statistics System maintained by the CDC National Center for Health Statistics. These data sets are derived from death certificate information reported directly to the National Center for Health Statistics, which processes, codes, and tabulates the data. At the time we downloaded the data sets, they included a cumulative count of confirmed COVID-19 deaths from February 2, 2020, through November 28, 2020, for each state: total, stratified by age, stratified by race, and stratified by age and race.

We calculated crude COVID-19 death rates for the non-Hispanic White and non-Hispanic Black population by dividing the total number of deaths among that racial group by the population of the racial group. We calculated age-standardized rates using direct standardization with the standard population being the age/race distribution of the USA as a whole in 2019. We standardized death rates using six age groups: 0–29, 30–49, 50–64, 65–74, 75–84, and 85+. We chose these age categories to optimize the balance between having enough age strata to generate age-adjusted estimates and having too narrow age strata such that there were missing data and therefore precluding us from generating age-adjusted estimates for certain states. Using these categories, we were able to generate age-adjusted COVID-19 death rates for both the non-Hispanic White and non-Hispanic Black populations in 35 of the 50 states (Table 1). There were missing data for deaths of non-Hispanic Black people in some age strata because the CDC suppresses any cell counts less than 10. The 15 excluded states were all less populated states with small Black populations. Thus, their exclusion should not substantially alter conclusions regarding the relationship between structural racism on the vast majority of Black communities in the USA. In fact, the 35 states included in the study account for 97.8% of the national non-Hispanic Black population. Population data by age, race, and state were obtained from the 2019 American Community Survey.

**Table 1:** Crude and age-adjusted racial/ethnic disparities in COVID-19 mortality rates between the non-Hispanic Black and non-Hispanic White populations (*N* = 35 states)

	Crude			Age-Adjusted			
State	Black death rate	White death rate	Disparity ratio	Black death rate	White death rate	Disparity ratio	Racism index
Alabama	114.0	88.2	1.3	142.7	73.3	1.9	35.0
Arizona	62.4	66.5	0.9	95.9	44.1	2.2	27.4
Arkansas	80.6	86.0	0.9	110.2	69.9	1.6	34.3
California	60.9	39.5	1.5	66.7	27.6	2.4	53.1
Colorado	71.6	43.0	1.7	112.8	42.3	2.7	52.7
Connecticut	194.8	144.0	1.4	258.0	94.4	2.7	60.3
Florida	105.3	79.6	1.3	139.2	46.0	3.0	35.2
Georgia	90.8	73.3	1.2	137.5	66.1	2.1	34.9
Illinois	129.3	79.8	1.6	151.5	61.7	2.5	61.0
Indiana	119.1	81.9	1.5	176.3	72.8	2.4	41.9
Iowa	70.7	88.4	0.8	174.1	70.1	2.5	52.4
Kansas	88.6	58.2	1.5	125.1	47.1	2.7	48.5
Kentucky	67.1	48.5	1.4	93.7	45.3	2.1	25.6
Louisiana	167.0	105.5	1.6	219.3	92.2	2.4	41.1
Maryland	108.9	71.1	1.5	136.8	53.9	2.5	46.7
Massachusetts	149.3	141.5	1.1	215.9	104.7	2.1	51.5
Michigan	193.9	62.9	3.1	230.0	51.4	4.5	51.9
Minnesota	61.7	63.3	1.0	163.5	51.5	3.2	62.6
Mississippi	142.8	111.2	1.3	194.1	92.3	2.1	39.4
Missouri	95.8	71.1	1.3	126.6	59.6	2.1	39.3
Nebraska	59.7	62.5	1.0	106.4	51.9	2.1	58.2
Nevada	83.4	58.1	1.4	113.8	44.6	2.6	31.1
New Jersey	238.8	170.5	1.4	277.1	118.7	2.3	66.3
New York	288.4	145.3	2.0	308.8	94.4	3.3	58.5
North Carolina	43.7	27.0	1.6	54.7	22.5	2.4	38.6
Ohio	70.5	56.3	1.3	87.4	46.6	1.9	48.6
Oklahoma	47.3	60.9	0.8	66.6	48.5	1.4	37.3
Pennsylvania	135.3	80.4	1.7	168.2	57.0	3.0	53.2
Rhode Island	122.5	137.2	0.9	168.2	93.2	1.8	50.6
South Carolina	116.9	75.2	1.6	141.3	62.7	2.3	43.4
Tennessee	82.7	62.9	1.3	114.6	55.0	2.1	33.7
Texas	68.7	62.3	1.1	105.5	53.9	2.0	34.7
Virginia	68.5	47.0	1.5	84.5	40.0	2.1	42.8
Washington	25.7	32.1	0.8	44.1	27.6	1.6	33.0
Wisconsin	78.3	61.9	1.3	143.9	49.8	2.9	72.3
**United States**	**108.9**	**69.0**	**1.6**	**149.3**	**55.1**	**2.7**	**------**

The age-adjusted mortality rates were calculated using direct standardization to the 2019 US population

#### Main Predictor Variable

The main predictor variable was the state racism index, which we developed and validated in previous research exploring the relationship between structural racism and racial disparities in fatal police shootings [49, 52]. The state racism index was adapted from previous scales developed by Wallet Hub [53] and by Lukachko et al. [54] and was shown to significantly predict differences between states in the Black–White disparity in fatal police shooting rates of unarmed victims [49]. The state racism index was listed in the Ford et al. book entitled “Racism: Science & Tools for the Public Health Professional” (see Appendix B: Selected Measures of Racism) [55]. The dimensions in the state racism index are identical to those used by Tan et al. [42] in their study of structural racism and county-level COVID-19 death rates (residential segregation, incarceration, economic status, and employment status), except that we additionally consider racial disparities in education. Our methods are also similar to those of Chambers et al. [56], who examined the relationship between structural racism at the county level and birth outcomes, and to those of Liu et al. [57], who measured structural racism at the county level and related it to maternal morbidity. We consulted the Groos et al. review of methods used to quantify structural racism [58] and confirmed that our approach is consistent with that used in multiple other studies.

Details of the state racism index have been presented elsewhere [49]. Briefly, the index measures structural racism at the state level across five dimensions: (1) residential segregation; (2) incarceration; (3) educational attainment; (4) economic indicators; and (5) employment status. The residential segregation dimension consists of two components: (a) the index of dissimilarity, a measure of the differential distribution of two population groups; and (b) the isolation index, a measure of the spatial isolation of one racial group from another. The incarceration dimension has one component: the ratio of the incarceration rate of Black people to the incarceration rate of White people. The educational attainment dimension has one component: the ratio of the proportion of Black people without a college education to the proportion of White people without a college education. The economic dimension consists of three components: (a) the ratio of the proportion of Black people in rental housing to that of White people in rental housing; (b) the ratio of the Black poverty rate to the White poverty rate; and (c) the ratio of median household income for the White population to median household income for the Black population. The employment dimension has two components: (a) the ratio of the Black unemployment rate to the White unemployment rate and (b) the ratio of the Black labor nonparticipation rate to the White labor nonparticipation rate. For each measure, the values across the 50 states are converted into a scale from 0 to 100. The components are then averaged to obtain a dimension score for each state. Finally, the scores for the five dimensions are averaged to yield the final state racism index. We constructed the state racism index using data from the 2019 American Community Survey (education, economics, and employment), 2018 national prisoner data from the Bureau of Justice Statistics (incarceration rates) [59], and the 2010 Decennial Census (segregation measures). The index of dissimilarity and the isolation index were calculated for the non-Hispanic White and non-Hispanic Black populations using the Census block as the lower level geographic unit. Details are shown in Appendix Table 5.

#### Potential Mediating Variables

Although our primary objective was to explore the relationship between structural racism and Black–White disparities in COVID-19 mortality, a secondary aim was to examine potential mediating factors and to investigate whether or not these factors completely explained any observed association. Therefore, we collected data on racial disparities in several areas that could potentially explain racial differences in COVID-19 effects. We were guided by the work of Garcia et al. [60], who—in an article on structural racism and the disproportionate impact of the pandemic on Black and Latinx adults—identified three mechanisms through which structural racism may operate to increase the burden of COVID-19 among these racial/ethnic groups: (1) increased risk of exposure (e.g., disproportionate proportion of the population in high-contact essential jobs); (2) weathering processes (disproportionate presence of pre-existing health conditions that may exacerbate the impact of COVID-19); and (3) disparities in health care access and quality. We selected variables in each of these three categories that are available at the state level and by race/ethnicity.

##### Differential Exposure Due to Occupation

We used race-and state-specific occupational data from the 2019 American Community Survey [61] to derive two estimates of Black–White disparities in occupations that may disproportionately expose workers to COVID-19. First, we calculated the proportion of workers for each racial/ethnic group in “exposed” jobs. Following the classification suggested by Baker et al. [62], exposed workers included those in health care occupations and those in protective service occupations. Second, we calculated the proportion of workers in “essential” jobs. The categories included here were health care occupations, protective service occupations, food preparation and serving, cleaning and maintenance, personal care and services, construction, repair, production, and transportation and material moving. For each measure, we operationalized the racial disparity as the ratio of the proportion of Black workers in those occupations to the proportion of White workers in those occupations. Details are shown in Appendix Table 6.

##### Differential Severity of Disease Due to Co-morbidities

Using the 2019 Behavioral Risk Factor Surveillance System (BFSSS) surveys [63], we calculated the proportion of adults in each state who self-reported the presence of each of the following conditions: hypertension, asthma, heart attack, angina, diabetes, obesity, kidney disease, cancer, stroke, and chronic obstructive lung disease (COPD). We also calculated the proportion of adults who reported any one or more of these conditions and the proportion who reported two or more of these conditions. These calculations were performed separately for the non-Hispanic White population and the non-Hispanic Black population. We estimated the Black–White racial disparity in these co-morbidities by dividing the proportion of Black adults with the condition by the proportion of White adults with the condition.

##### Differences in Medical Care Due to Insurance Coverage Disparities

Using the 2019 Behavioral Risk Factor Surveillance System (BFSSS) surveys [63], we calculated the race-specific proportion of adults in each state who reported not having health insurance and the proportion who reported not being able to afford medical care at some point in the past year. The Black–White disparity in health care access was defined as the ratio of non-Hispanic Black adults who were not insured or could not afford medical care divided by the proportion of non-Hispanic White adults who were uninsured or could not afford medical care.

### Data Analysis

We began by calculating crude COVID-19 death rates for the non-Hispanic White and non-Hispanic Black population in each state. We then calculated race-specific, age-adjusted COVID-19 death rates and compared these to the unadjusted rates. Next, we conducted linear regression analyses to examine the relationship between the state racism index and differences in the magnitude of the Black–White disparity in age-adjusted COVID-19 death rates across states. We then examined the relationship between each of the three mediating variables and the racial disparity in death rates. Finally, we explored whether including the mediating variables in the same linear regression would nullify the relationship between the state racism index and any observed racial disparities in COVID-19 mortality. Because of the multicollinearity between many of these predictor variables (Appendix Table 7), we examined variance inflation factors for these multiple linear regressions and did not draw any inferences from analyses unless all variance inflation factors were below 3.

### Sensitivity Analysis

Because the racial disparity in incarceration rates is one component of the state racism index and differences in incarceration rates have been shown to have a direct effect on the likelihood of COVID-19 exposure [64], we repeated the analyses with a recalculated state racism index that did not include the incarceration dimension. The finding of an association between this revised racism index and racial disparities in COVID-19 mortality would suggest that any observed relationship between structural racism and differences in the racial disparity in COVID-19 mortality across states was not being driven completely by racial differences in incarceration rates.

## Results

### Descriptive Results

For all 35 states, the Black–White disparity in COVID-19 mortality rates was substantially greater when examining age-adjusted rates compared to crude rates (Table 1). This shows that relying on the crude death rates results in a marked underestimation of the magnitude of the racial disparity in COVID-19 mortality between the Black and White populations. For example, in eight states, the crude death rates suggested that there was either no disparity or that the Black population was dying from COVID-19 at a lower rate than the White population. After direct age standardization, it became apparent that the COVID-19 death rate for the Black population was higher than that for the White population in all eight states, with the rate ratio ranging from 1.4 to 3.2. Perhaps the most dramatic difference was in Minnesota, where the crude death rates were essentially the same for the Black and White populations, but the age-adjusted rates revealed a marked disparity, with Black Minnesotans dying at a rate 3.2 times greater than White Minnesotans. For the USA overall, the ratio of Black to White crude COVID-19 death rates was 1.6, while the ratio of age-adjusted death rates was 2.7.

The age-adjusted death rate disparity ratios ranged from a low of 1.4 in Oklahoma to a high of 4.5 in Michigan (Table 1). The five states with the highest disparity ratios were Michigan (4.5), New York (3.3), Minnesota (3.2), Florida (3.0), and Pennsylvania (3.0). The five states with the lowest disparity ratios were Oklahoma (1.4), Washington (1.6), Arkansas (1.6), Rhode Island (1.8), and Ohio (1.9).

Among the 35 states, the state structural racism index ranged from a low of 25.6 in Kentucky to a high of 72.3 in Wisconsin (Table 1). The five states with the highest racism index were all located in the Midwest or Northeast: Wisconsin (72.3), New Jersey (66.3), Minnesota (62.6), Illinois (61.0), and Connecticut (60.3). The five states with the lowest racism index were all located in the Southeast or West: Kentucky (25.6), Arizona (27.4), Nevada (31.1), Washington (33.0), and Tennessee (33.7). The overall racism index for each state along with the component indices are displayed in Appendix Table 8.

### Analytic Results

**Fig. 1:**
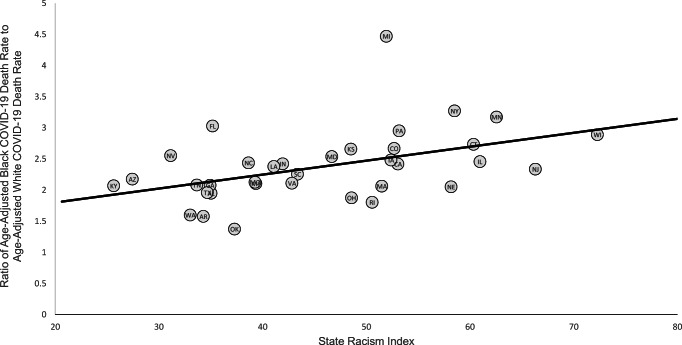
Ratio of Black age-adjusted COVID-19 death rate to White age-adjusted COVID-19 death rate as a function of the state racism index. The racism index is on a scale of 0 to 100 with higher numbers indicating a greater level of structural racism. Mortality rates are per 100,000 population

**Fig. 2:**
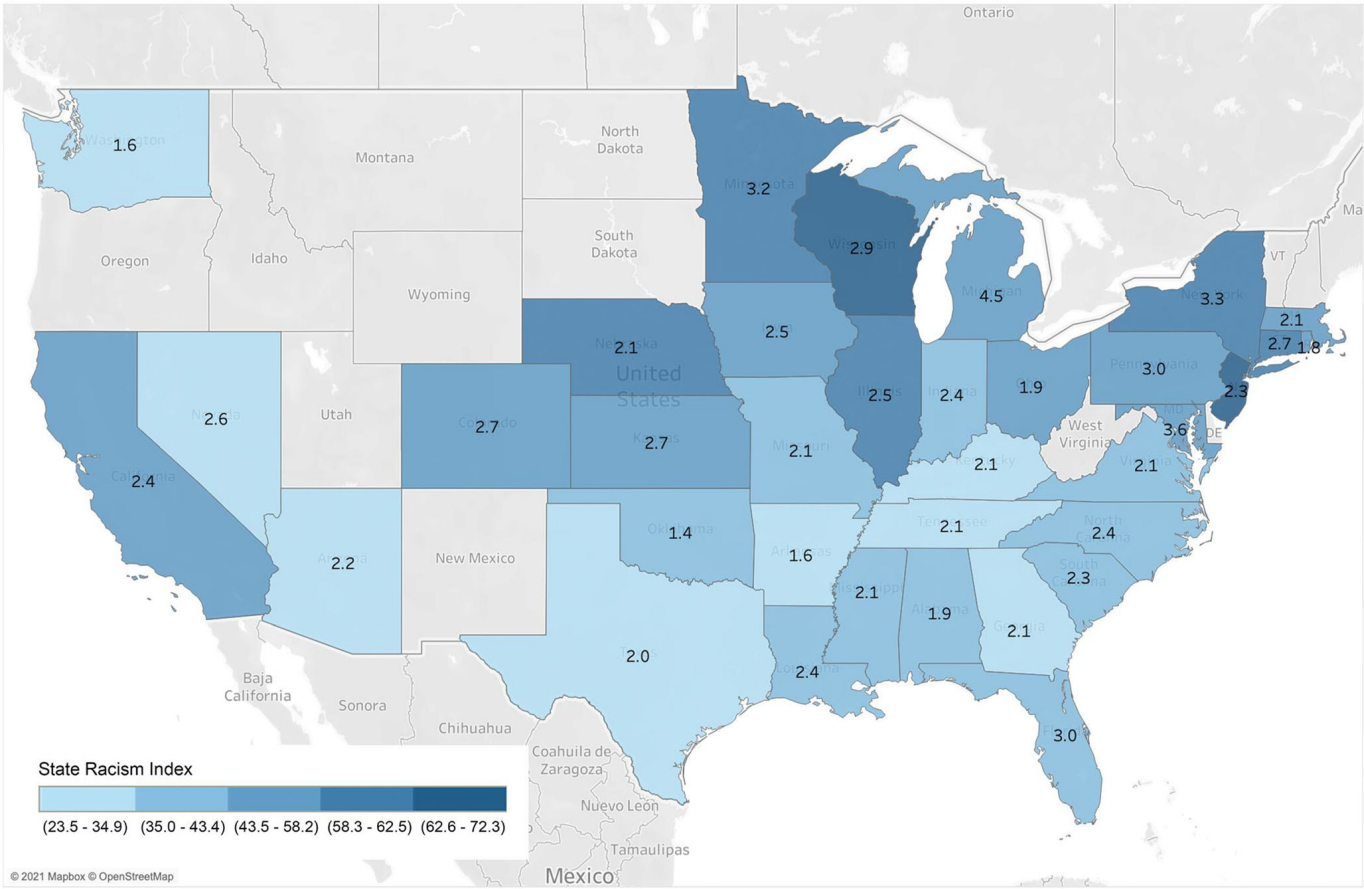
Heat map showing the state structural racism index in gradations of color with the ratio of the age-adjusted Black COVID-19 mortality rate to the age-adjusted White COVID-19 mortality rate shown in text in the middle of each state

The five states with the highest Black–White disparity in COVID-19 mortality rates had an average state structural racism index of 52.3, compared to 40.8 for the five states with the lowest disparity (Table 1). The five states with the highest structural racism indices had an average disparity ratio of 2.7, compared to 2.1 for the five states with the lowest racism indices. A scatterplot of this relationship for all 35 states showed a pattern of increasing racial disparities as the state structural racism index increased (Fig. 1). A heat map also showed a pattern of higher racism indices being associated with greater Black–White disparities in age-adjusted COVID-19 death rates (Fig. 2). The linear regression indicated that for each standard deviation increase in the state racism index, the COVID-19 death rate disparity ratio increased by 0.26 (95% CI, 0.08 to 0.44, *P* = 0.006) (Table 2). Each of the five separate components of the state racism index was positively associated with the magnitude of the Black–White disparity in COVID-19 death rates, although this relationship was statistically significant only for the incarceration index and the economic index.

**Table 2:** Results of linear regression showing coefficients representing the change in the ratio of Black to White age-adjusted COVID-19 death rates for each standard deviation increase in the state racism index and its components, 95% confidence intervals (CI), and *P* values in bivariate models (*N* = 35 states)

Racism index	Regression coefficient	95% CI	*P* value
Overall racism index	0.26	(0.08, 0.44)	0.006
Segregation index	0.19	(−0.07, 0.45)	0.140
Incarceration index	0.18	(0.02, 0.35)	0.034
Education index	0.19	(−0.01, 0.40)	0.067
Economic index	0.25	(0.01, 0.50)	0.041
Employment index	0.15	(−0.07, 0.37)	0.178

The regression coefficient shows the change in the ratio of Black to White COVID-19 death rates for each standard deviation increase in the relevant index

In the multivariate analyses, the addition of racial disparities in occupational exposure, comorbidities, and health care insurance or affordability did not substantially alter the relationship between the state racism index and the Black–White racial disparity in COVID-19 mortality (Table 3). In the final model, which controlled for all three of the potential mediating factors, the regression coefficient for the state racism index was 0.29 (95% CI, 0.04–0.54; *P* = 0.022).

**Table 3:** Results of linear regression showing coefficients representing the change in the ratio of Black to White age-adjusted COVID-19 death rates for each standard deviation increase in the state racism index, 95% confidence intervals (CI), *P* values, and variance inflation factors (VIF) in multivariate models (*N*=35 states)

Variables in model	Regression coefficient	95% CI	*P* value	VIF
*Model 1*				
State racism index	0.26	(0.04, 0.49)	0.023	1.49
Black–White disparity in exposed jobs	0.00	(−0.24, 0.23)	0.967	1.49
*Model 2*				
State racism index	0.30	(0.09, 0.50)	0.006	1.26
Black–White disparity in essential jobs	−0.25	(−0.87, 0.37)	0.416	1.26
*Model 3*				
State racism index	0.26	(0.07, 0.45)	0.008	1.07
Black–White disparity in any comorbidity	−0.02	(−0.48, 0.44)	0.925	1.07
*Model 4*				
State racism index	0.42	(0.16, 0.68)	0.002	2.13
Black–White disparity in health insurance coverage	−0.28	(−0.61, 0.06)	0.099	2.13
*Model 5*				
State racism index	0.30	(0.11, 0.49)	0.003	1.13
Black–White disparity in inability to afford health care	−0.18	(−0.49, 0.13)	0.241	1.13
*Model 6*				
State racism index	0.29	(0.04, 0.54)	0.022	1.79
Black–White disparity in exposed jobs	0.02	(−0.24, 0.28)	0.854	1.74
Black–White disparity in any comorbidity	−0.05	(−0.55, 0.45)	0.839	1.21
Black–White disparity in inability to afford health care	−0.19	(−0.53, 0.14)	0.240	1.20

The regression coefficient shows the change in the ratio of Black to White COVID-19 death rates for each standard deviation increase in the independent variable

### Sensitivity Analysis

We reran the regressions using a state racism index that did not include the incarceration component. In the bivariate analysis, the regression coefficient associated with a one standard deviation increase in the state racism index was 0.31 instead of 0.26 and remained statistically significant. In the multivariate analysis, the regression coefficient associated with a one standard deviation increase in the state racism index was 0.37 instead of 0.29 and remained statistically significant.

## Discussion

To the best of our knowledge, this is the first paper to quantify the Black–White disparity in COVID-19 mortality at the state level using directly standardized age-adjusted death rates. It is also the first paper to explicitly model the Black–White disparity in COVID-19 mortality at the state level as a function of an empirical measure of structural racism. We found that relying upon crude death rate ratios resulted in a substantial underestimation of the true magnitude of the disparity between Black and White COVID-19 mortality rates. We also found that there is a robust relationship between the state structural racism index and the magnitude of the Black–White disparity in COVID-19 mortality rates. Each standard deviation increase in the racism index was associated with an increase of 0.26 in the ratio of directly age-adjusted COVID-19 mortality rates among the non-Hispanic Black compared to the non-Hispanic White population.

The importance of direct age standardization in examining racial/ethnic disparities in COVID-19 mortality is illustrated by a comparison of our estimate of the Black–White death rate ratio in Minnesota (3.2) to that in previous studies. Two studies that relied upon crude death rates reported ratios of 1.45 [40] and 1.7 [38], and one study that relied upon indirectly age-adjusted death rates reported a ratio of 1.4 [39]. The COVID Racial Data Tracker (as of February 2021), which relies upon crude death rate estimates, reported no racial/ethnic disparity between non-Hispanic Black and non-Hispanic White COVID-19 mortality rates in Minnesota [34]. Finally, the APM Research Lab, which uses indirect age standardization, reported a Black–White death rate ratio of 2.1 for Minnesota as of February 2021 [33]. We found a similar pattern of underestimation of the Black–White racial disparity in COVID-19 mortality rates for the other states in our study as well.

The most likely explanation for the discrepancy between the crude and age-adjusted estimates of the Black–White disparity in COVID-19 mortality is that life expectancy is lower for Black people compared to White people [65]; therefore, the age distribution for the Black population is skewed toward lower ages. Since lower age correlates with lower COVID-19 mortality risk, the crude death rate ratios provide an underestimate of the true racial disparities. The lower life expectancy for Black people is itself associated with structural racism; thus, ironically, it is structural racism that explains why the age-adjusted COVID-19 death rate ratios are much higher than the crude ones. In fact, in our data set, the absolute magnitude of the difference between the crude and adjusted death rate ratios is strongly correlated with the state racism index (r = 0.45).

While many previous studies have documented the Black–White racial disparity in COVID-19 mortality, this is the first to explicitly model the differences in the level of this racial disparity between states as an outcome variable in an attempt to find factors that explain what one might call the “disparity in the racial disparity” across states. This “disparity in disparities” is quite striking. For example, in Michigan, a Black person is 4.5 times more likely to die of COVID-19 than a White person, while in Oklahoma, a Black person is just 1.4 times more likely to die of COVID-19 than a White person. We have shown that one factor associated with the magnitude of this racial disparity in a state is the level of structural racism in that state. The highest Black–White disparities in COVID-19 mortality rates were observed in the upper Midwest and the Northeast, precisely those regions which have the highest structural racism indices.

Although six other studies [42–47] found a relationship between measures of structural racism and racial disparities in COVID-19 mortality at the county level, this paper documents such a relationship at the state level. This finding is important because it is at the state level that most power resides to enact or refine laws related to public health and safety that could directly impact these observed disparities [48].

Notably, the robust relationship between the state structural racism index and the state-level Black–White disparity in COVID-19 mortality persisted even after controlling for racial differences in the proportion of the population with various comorbidities, the proportion of workers in essential jobs, or those with high levels of exposure, and the proportion of the population without health insurance or without the ability to pay for health care. This finding is significant because it suggests that the explanation for the observed disparities goes beyond differences in health care access, pre-existing health conditions, and the likelihood of exposure. This implies that intervening at the level of the individual (i.e., medical treatment or reducing exposure) or at the level of the health care system (i.e., improving health care access) is not adequate to eliminate racial disparities in COVID-19-related mortality. As structural racism in the USA has been a 400-year process of deeply rooted racist practices and sustained inequity, it is important to recognize that changes in underlying economic and political power structures are necessary in order to genuinely abridge racial disparities. It is still important to address downstream consequences of structural racism, such as inequities in health conditions, access, and exposure; however, interventions must dismantle structural racism directly since it is the upstream process, embedded within the culture of the USA, which has created and sustained those inequities. This research suggests that structural racism itself should be considered an underlying cause of COVID-19 racial/ethnic disparities. Thus, it provides empirical data to support the view that structural or institutionalized racism must be viewed as the fundamental or root cause of COVID-19-related racial health disparities [60, 66–74].

## Limitations

The primary limitation of this study is the limited availability of age-specific and race/ethnicity-specific COVID-19 mortality data in several states. This forced us to limit our analysis to 35 states for which sufficient data were provided to generate directly age-standardized death rates. Therefore, our results cannot necessarily be generalized to all 50 states. Similarly, there were data limitations in calculating the racism indices for several states. However, since these were the same states for which there were limited mortality data, it did not result in our having to drop any further states from the analysis. A second important limitation is that in controlling for factors that are directly related to COVID-19 mortality risk, our analysis was subject to the problem of multicollinearity, as these factors tend to be correlated with structural racism as well. It is reassuring that the variance inflation factors were generally less than two, a level that would indicate a multicollinearity problem. Additionally, since COVID-19 is not well understood, there could be unknown factors that confound the relationship between structural racism and disease mortality rates. Third, our state racism index is just one of many that have been used to quantify structural racism for empirical analyses. Although we have used and validated this measure previously, we acknowledge that there is no singular approach to operationalize such a complex phenomenon. While the intent of this paper was specifically to examine Black–White differences in COVID-19 mortality, it is important to acknowledge that other racial-ethnic groups, including Latinx, Asian American, and Indigenous populations, are also experiencing a disproportionate burden of disease and death, and the causes of those disparities deserve attention as well. There is evidence that the nature and effects of racial residential segregation and structural racism are different for various racial/ethnic groups [75]. As Gee emphasizes, “one should not assume a ‘one size fits all’ conceptualization of contextual effects” [75 , p. 621]. Future research should explore both common and group-specific mechanisms by which structural racism creates racial disparities in COVID-19 morbidity and mortality.

## Conclusion

Despite these limitations, this paper demonstrates that actual disparities in COVID-19 mortality between the non-Hispanic Black and non-Hispanic White populations are greater than what has previously been reported in studies that failed to consider the race/ethnicity-specific age distribution of the population. Furthermore, this research documents that there are marked differences in the magnitude of the Black–White disparity in COVID-19 mortality across states and that these differences are associated with the level of structural racism in those states. We conclude that structural racism must now be considered a root cause of the observed Black–White disparity in COVID-19 mortality. Our research suggests that the dismantling of long-standing systems of racial oppression is critical to adequately address both the downstream and upstream causes of racial inequities in the disease burden of COVID-19. As vaccination is now being implemented, there is a danger that inequities in vaccine distribution may further exacerbate racial/ethnic disparities in COVID-19-related morbidity and mortality. Therefore, prioritization of Black communities for vaccine distribution is essential in the nation’s COVID-19 recovery plan.

## Supplementary Information

ESM 1 (DOCX 34 kb)

## Data Availability

The database produced in this research project is available from the corresponding author.

## Abbreviations

ACS: American Community Survey
APM: American Public Media
BRFSS: Behavioral Risk Factor Surveillance System
CDC: Centers for Disease Control and Prevention
CI: confidence interval
COPD: chronic obstructive pulmonary disease
COVID-19: coronavirus disease 2019
NCHS: National Center for Health Statistics
SRR: standardized rate ratio

## References

[1] Johns Hopkins University & Medicine. Coronavirus resource center. Baltimore, MD: JHU.edu; 2021 [Available from: https://coronavirus.jhu.edu/]. Accessed 25 Jan 2021.

[2] Bassett MT, Chen JT, Krieger N (2020). Variation in racial/ethnic disparities in COVID-19 mortality by age in the United States: a cross-sectional study. PLoS Med.

[3] Lee IJ, Ahmed NU. The devastating cost of racial and ethnic inequity in the COVID-19 pandemic. J Natl Med Assoc. 2021. 10.1016/j.jnma.2020.11.015 Available from: Accessed 26 Jan 2021. 10.1016/j.jnma.2020.11.015 33339615

[4] Tirupathi R, Muradova V, Shekhar R, Salim SA, Al-Tawfiq JA, Palabindala V (2020). COVID-19 disparity among racial and ethnic minorities in the US: a cross sectional analysis. Travel Med Infect Dis.

[5] Raine S, Liu A, Mintz J, Wahood W, Huntley K, Haffizulla F (2020). Racial and ethnic disparities in COVID-19 outcomes: social determination of health. Int J Environ Res Public Health.

[6] Figueroa JF, Wadhera RK, Mehtsun WT, Riley K, Phelan J, Jha AK (2021). Association of race, ethnicity, and community-level factors with COVID-19 cases and deaths across U.S. counties. Healthcare.

[7] Cyrus E, Clarke R, Hadley D, Bursac Z, Trepka MJ, Devieux JG, et al. The impact of COVID-19 on African American communities in the United States. medRxiv. 2020. 10.1101/2F2020.05.15.20096552 Available from: Accessed 26 Jan 2021. 10.1089/heq.2020.0030 PMC7702977 33269331

[8] Adhikari S, Pantaleo NP, Feldman JM, Ogedegbe O, Thorpe L, Troxel AB (2020). Assessment of community-level disparities in Coronavirus disease 2019 (COVID-19) infections and deaths in large US metropolitan areas. JAMA Netw Open.

[9] Khanijahani A. Racial, ethnic, and socioeconomic disparities in confirmed COVID-19 cases and deaths in the United States: a county-level analysis as of November 2020. Ethn Health. 2020. 10.1080/13557858.2020.1853067 Available from: Accessed 26 Jan 2021. 10.1080/13557858.2020.1853067 33334160

[10] Mahajan UV, Larkins-Pettigrew M (2020). Racial demographics and COVID-19 confirmed cases and deaths: a correlational analysis of 2886 US counties. J Public Health.

[11] Kim SJ, Bostwick W (2020). Social vulnerability and racial inequality in COVID-19 deaths in Chicago. Health Educ Behav.

[12] Millett GA, Jones AT, Benkeser D, Baral S, Mercer L, Beyrer C, Honermann B, Lankiewicz E, Mena L, Crowley JS, Sherwood J, Sullivan PS (2020). Assessing differential impacts of COVID-19 on black communities. Ann Epidemiol.

[13] Figueroa JF, Wadhera RK, Lee D, Yeh RW, Sommers BD (2020). Community-level factors associated with racial and ethnic disparities in COVID-19 rates in Massachusetts. Health Aff.

[14] Hamman MK (2021). Disparities in COVID-19 mortality by county racial composition and the role of spring social distancing measures. Econ Hum Biol.

[15] Akanbi MO, Rivera AS, Akanbi FO, Shoyinka A. An ecologic study of disparities in COVID-19 incidence and case fatality in Oakland County, MI, USA, during a state-mandated shutdown. J Racial Ethn Health Disparities. 2020. 10.1007/s40615-020-00909-1 Available from: Accessed 26 Jan 2021. 10.1007/s40615-020-00909-1 PMC7595050 33124003

[16] Strully K, Yang T-C, Liu H. Regional variation in COVID-19 disparities: connections with immigrant and Latinx communities in U.S. counties. Ann Epidemiol. 2020. 10.1016/j.annepidem.2020.08.016 Available from: Accessed 26 Jan 2021. 10.1016/j.annepidem.2020.08.016 PMC7485497 32927056

[17] Do DP, Frank R. Unequal burdens: assessing the determinants of elevated COVID-19 case and death rates in New York City’s racial/ethnic minority neighbourhoods. J Epidemiol Community Health. 2020. 10.1136/jech-2020-215280 Available from: Accessed 26 Jan 2021. 10.1136/jech-2020-215280 33122256

[18] Cheng KJG, Sun Y, Monnat SM (2020). COVID-19 death rates are higher in rural counties with larger shares of Blacks and Hispanics. J Rural Health..

[19] Feinhandler I, Cilento B, Beauvais B, Harrop J, Fulton L (2020). Predictors of death rate during the COVID-19 pandemic. Healthcare.

[20] Richmond HL, Tome J, Rochani H, Fung IC, Shah GH, Schwind JS (2020). The use of penalized regression analysis to identify county-level demographic and socioeconomic variables predictive of increased COVID-19 cumulative case rates in the state of Georgia. Int J Environ Res Public Health.

[21] DiMaggio C, Klein M, Berry C, Frangos S (2020). Black/African American communities are at highest risk of COVID-19: spatial modeling of New York City zip code-level testing results. Ann Epidemiol.

[22] Liao TF, De Maio F (2021). Association of social and economic inequality with coronavirus disease 2019 incidence and mortality across US counties. JAMA Netw Open.

[23] Karmakar M, Lantz PM, Tipirneni R (2021). Association of social and demographic factors with COVID-19 incidence and death rates in the US. JAMA Netw Open.

[24] Anaele BI, Doran C, McIntire R. Visualizing COVID-19 mortality rates and African-American populations in the USA and Pennsylvania. J Racial Ethn Health Disparities. 2021. 10.1007/s40615-020-00897-2 Available from: Accessed 11 Feb 2021. 10.1007/s40615-020-00897-2 PMC7872308 33565050

[25] Igedegbe G, Ravenell J, Adhikari S, Butler M, Cook T, Francois F (2020). Assessment of racial/ethnic disparities in hospitalization and mortality in patients with COVID-19 in New York City. JAMA Netw Open.

[26] Muñoz-Orice LS, Natinger AB, Rivera F, Hanson R, Gmehlin CG, Perez A (2020). Racial disparities in incidence and outcomes among patients with COVID-19. JAMA Netw Open.

[27] Vahidy FS, Nicolas JC, Meeks JR, Khan O, Pan A, Jones SL (2020). Racial and ethnic disparities in SARS-CoV2 pandemic: analysis of a COVID-19 observational registry for a diverse US metropolitan population. BMJ Open.

[28] Poulson M, Geary A, Annesi C, Allee L, Kenzik K, Sanchez S, et al. National disparities in COVID-19 outcomes between Black and White Americans. J Natl Med Assoc. 2020. 10.1016/j.jnma.2020.07.009 Available from: Accessed 26 Jan 2021. 10.1016/j.jnma.2020.07.009 PMC7413663 32778445

[29] Cromer SJ, Lakhani CM, Wexler DJ, Burnett-Bowie SM, Udler M, Patel CJ. Geospatial analysis of individual and community-level socioeconomic factors impacting SARS-CoV-2 prevalence and outcomes. medRxiv. 2020. 10.1101/2020.09.30.20201830 Available from: Accessed 26 Jan 2021.

[30] Wrigley-Field E, Garcia S, Leider JP, Robertson C, Wurtz R (2020). Racial disparities in COVID-19 and excess mortality in Minnesota. Socius.

[31] Wiley Z, Kubes JN, Cobb J, Jacob JT, Franks N, Plantinga L, et al. Age, comorbid conditions, and racial disparities in COVID-19 outcomes. J Racial Ethn Health Disparities. 2021. 10.1007/s40615-020-00934-0 Available from: Accessed 26 Jan 2021. 10.1007/s40615-020-00934-0 PMC7790329 33415702

[32] Ford T, Reber S, Reeves R (2020). Race gaps in COVID-19 are even bigger than they appear.

[33] APM Research Lab. The color of coronavirus: COVID-19 deaths by race and ethnicity in the U.S. St. Paul, MN: American Public Media; 2021 [Available from: https://www.apmresearchlab.org/covid/deaths-by-race]. Accessed 24 Jan 2021.

[34] COVID Tracking Project and the Boston University Center for Antiracist Research (2021). The COVID Racial Data Tracker.

[35] Abedi V, Olulana O, Avula V, Chaudhary D, Khan A, Shahjouei S, et al. Racial, economic and health inequality and COVID-19 infection in the United States. medRxiv. 2020. 10.1007/s40615-020-00833-4 Available from: Accessed 26 Jan 2021. 10.1007/s40615-020-00833-4 PMC7462354 32875535

[36] Anyane-Yeboa A, Sato T, Sakuraba A (2020). Racial disparities in COVID-19 deaths reveal harsh truths about structural inequality in America. J Intern Med.

[37] Parcha V, Malla G, Suri SS, Kaira R, Heindl B, Berra L (2020). Geographic variation of racial disparities in health and COVID-19 mortality. Mayo Clin Proc Inn Qual Out.

[38] Boserup B, McKenney M, Elkbuli A (2020). Disproportionate impact of COVID-19 pandemic on racial and ethnic minorities. Am Surg.

[39] Goldstein JR, Atherwood S. Improved measurement of racial/ethnic disparities in COVID-19 mortality in the United States. medRxiv. 2020. 10.1101/2020.05.21.20109116 Available from: Accessed 26 Jan 2021.

[40] Gross CP, Essien UR, Pasha S, Gross JR, Wang S, Nunez-Smith M (2020). Racial and ethnic disparities in population-level Covid-19 mortality. J Gen Intern Med.

[41] Curtin LR, Klein RJ. Direct standardization (age-adjusted death rates). Healthy People 2000 Statistical Notes. Hyattsville, MD: Centers for Disease Control and Prevention, National Center for Health Statistics; 1995 [Available from: https://www.cdc.gov/nchs/data/statnt/statnt06rv.pdf]. Accessed 26 Jan 2021. 11762384

[42] Tan SB, deSouza P, Raifman M. Structural racism and COVID-19 in the USA: a county-level empirical analysis. J Racial Ethn Health Disparities. 2021. 10.1007/s40615-020-00948-8 Available from: Accessed 26 Jan 2021. 10.1007/s40615-020-00948-8 PMC7815192 33469868

[43] Khanijahani A, Tomassoni L. Socioeconomic and racial segregation and COVID-19: concentrated disadvantage and Black concentration in association with COVID-19 deaths in the USA. J Racial Ethn Health Disparities. 2021. 10.1007/s40615-021-0965-1 Available from: Accessed 26 Jan 2021. 10.1007/s40615-021-00965-1 PMC7815201 33469872

[44] Cunningham GB, Wigfall LT (2020). Race, explicit racial attitudes, implicit racial attitudes, and COVID-19 cases and deaths: an analysis of counties in the United States. PLoS ONE.

[45] Li D, Gaynor SM, Quick C, Chen JT, Stephenson BJK, Coull BA, et al. Unraveling US national COVID-19 racial/ethnic disparities using county level data among 328 million Americans. medRxiv. 2020. 10.1101/2020.12.02.20234989 Available from: Accessed 26 Jan 2021.

[46] Yang T-C, Choi SE, Sun F. COVID-19 cases in US counties: roles of racial/ethnic density and residential segregation. Ethn Health. 2020. 10.1080/13557858.2020.1830036 Available from: Accessed 26 Jan 2021. 10.1080/13557858.2020.1830036 33059471

[47] Yu Q, Salvador CE, Melani I, Berg MK, Neblett EW, Kitayama S. Racial residential segregation and economic disparity jointly exacerbate COVID-19 fatality in large American cities. Ann N Y Acad Sci. 2021. 10.1111/nyas.14567 Available from: Accessed 2 Feb 2021. 10.1111/nyas.14567 PMC8013888 33521931

[48] American Bar Association. Two centuries of law guide legal approach to modern pandemic. 2020. Chicago, IL: ABA; 2020 [Available from: https://www.americanbar.org/news/abanews/publications/youraba/2020/youraba-april-2020/law-guides-legal-approach-to-pandemic/]. Accessed 25 Jan 2021.

[49] Mesic A, Franklin L, Cansever A, Potter F, Sharma A, Knopov A, Siegel M (2018). The relationship between structural racism and Black–White disparities in fatal police shootings at the state level. J Natl Med Assoc.

[50] Laurencin CT, McClinton A. The COVID-19 pandemic: a call to action to identify and address racial and ethnic disparities. J Racial Ethn Health Disparities. 2020:1–5. 10.1007/2Fs40615-020-00756-0 Available from: Accessed 8 Feb 2021. 10.1007/s40615-020-00756-0 PMC7166096 32306369

[51] Centers for Disease Control and Prevention. COVID-19 Death Data and Resources. Hyattsville, MD: National Center for Health Statistics; 2021. [Available from: https://www.cdc.gov/nchs/nvss/covid-19.htm]. Accessed 14 Jan 2021.

[52] Siegel M (2020). Racial disparities in fatal police shootings: an empirical analysis informed by critical race theory. Boston Univ Law Rev.

[53] Hub W (2017). 2017’s states with the most racial progress.

[54] Lukachko A, Hatzenbuehler ML, Keyes KM (2014). Structural racism and myocardial infarction in the United States. Soc Sci Med.

[55] Ford CL, Griffith DM, Bruce MA, Gilbert KL (2019). Racism: science & tools for the public health professional.

[56] Chambers BD, Erausquin JT, Tanner AE, Nichols TR, Brown-Jeffy S (2018). Testing the association between traditional and novel indicators of county-level structural racism and birth outcomes among Black and White women. J Racial Ethn Disparities.

[57] Liu SY, Fiorentini C, Bailey Z, Huynh M, McVeigh K, Kaplan D (2019). Structural racism and severe maternal morbidity in New York State. Clin Med Insights Womens Health.

[58] Groos M, Wallace M, Hardeman R, Theall K (2018). Measuring inequity: a systematic review of methods used to quantify structural racism. J Health Dispar Res Pract.

[59] Bureau of Justice Statistics (2020). Prisoners in 2019.

[60] Garcia MA, Homan PA, Garcia C, Brown TH. The color of COVID-19: structural racism and the disproportionate impact of the pandemic on the older Black and Latinx adults. J Gerontol B Psychol Sci Soc Sci. 2020. 10.1093/geronb/gbaa114 Available from: Accessed 26 Jan 2021. 10.1093/geronb/gbaa114 PMC7454923 32756973

[61] American Community Survey. Sex by occupation for the civilian employed population 16 years and over, by race/ethnicity, 2019. Table B24010. Washington, DC: United States Census Bureau; 2019.

[62] Baker MG, Peckham TK, Seixas NS (2020). Estimating the burden of United States workers exposed to infection or disease: a key factor in containing risk of COVID-19 infection. PLoS ONE.

[63] Centers for Disease Control and Prevention (2019). BRFSS Survey Data and Documentation.

[64] Nowotny KM, Bailey Z, Brinkley-Rubinstein L (2021). The contribution of prisons and jails to US racial disparities during COVID-19. Am J Public Health..

[65] Bharmal N, Tseng C-H, Kaplan R, Wong MD (2012). State-level variations in racial disparities in life expectancy. Health Serv Res.

[66] Churchwell K, Elkind MSV, Benjamin RM, Carson AP, Chang EK, Lawrence W (2020). Call to action: structural racism as a fundamental driver of health disparities. A presidential advisory from the American Heart Association. Circulation.

[67] Yaya S, Yeboah H, Charles CH, Out A, Labonte R (2020). Ethnic and racial disparities in COVID-19-related deaths: counting the trees, hiding the forest. BMJ Global Health.

[68] Gaynor TS, Wilson ME (2020). Social vulnerability and equity: the disproportionate impact of COVID-19. Public Adm Rev.

[69] Ford CL (2020). Commentary: addressing inequities in the era of COVID-19. The pandemic and the urgent need for critical race theory. Family Community Health.

[70] Maness SB, Merrell L, Thompson EL, Griner SB, Kline N, Wheldon C (2021). Social determinants of health and health disparities: COVID-19 exposures and mortality among African American people in the United States. Public Health Rep.

[71] Egede LE, Walker RJ (2020). Structural racism, social risk factors, and Covid-19—a dangerous convergence for Black Americans. N Engl J Med.

[72] Milner A, Franz B, Braddock IIJH (2020). We need to talk about racism—in all of its forms—to understand COVID-19 disparities. Health Equity.

[73] Kullar R, Marcelin JR, Swartz TH, Piggott DA, Macias Gil R, Mathew TA, Tan T (2020). Racial disparity of coronavirus disease 2019 in African American communities. J Infect Dis.

[74] Poteat T, Millett GA, Nelson LE, Beyrer C (2020). Understanding COVID-19 risks and vulnerabilities among black communities in America: the lethal force of syndemics. Ann Epidemiol.

[75] Gee GC (2002). A multilevel analysis of the relationship between institutional and individual racial discrimination and health status. Am J Public Health.

