# Supplementary material for: Actual Racial/Ethnic Disparities in COVID-19 Mortality for the Non-Hispanic Black Compared to Non-Hispanic White Population in 35 US States and Their Association with Structural Racism
Source: J Racial Ethn Health Disparities. 2021 Apr 27;9(3):886–98. doi: 10.1007/s40615-021-01028-1 (PMC8077854; doi:10.1007/s40615-021-01028-1)
Supplement: Supplementary file 1 — (DOCX 34 kb) [file 40615_2021_1028_MOESM1_ESM.docx]

**Appendix**

**Article title:** Actual Racial/Ethnic Disparities in COVID-19 Mortality for the Non-Hispanic Black Compared to Non-Hispanic White Population in 35 US States and their Association with Structural Racism

**Journal:** Journal of Racial and Ethnic Health Disparities

**Authors:** Michael Siegel, Isabella Critchfield-Jain, Matthew Boykin, and Alicia Owens

**Corresponding author:** Dr. Michael Siegel, Boston University School of Public Health; mbsiegel@bu.edu

**Appendix Table 1** Summary of previous studies that presented state-specific estimates of COVID-19 mortality rates among the non-

Hispanic Black and non-Hispanic White or Black and White populations

| **Study** | **Description** | **Age standardization** |
| --- | --- | --- |
| Parcha et al., 2020^36^ | Analyzes ratio of crude COVID-19 mortality rates for the Black compared to White population (regardless of ethnicity) for 40 states using data from state websites as of August 16, 2020 | None |
| Baserup et al., 2020^37^ | Presents crude COVID-19 death rates by state for non-Hispanic Black and non-Hispanic White population in 44 states using COVID-19 Tracking Project data as of July 15, 2020 | None |
| Gross et al., 2020^39^ | Presents ratio of crude COVID-19 mortality rates for non-Latinx Black vs. non-Latinx White population in 27 states using data from state websites as of April 21, 2020 | Indirect |
| Goldstein and Atherwood, 2020^38^ | Presents ratio of age- and place-adjusted COVID-19 mortality rates for non-Hispanic Black compared to non-Hispanic White population in 32 states using CDC provisional COVID-19 death counts as of May 13, 2020 | Indirect |
| APM Research Lab^32^ | Website presents periodically updated age-adjusted COVID-19 death rates for non-Hispanic Black and non-Hispanic White populations in 39 states using both state websites and CDC provisional COVID-19 death counts | Indirect |

**Appendix Table 2** Definitions, data sources, and methods for calculation of the state racism index, 2019

| **Dimension** | **Measure** | **Description** | **Data source** |
| --- | --- | --- | --- |
| Exposure risk | Dissimilarity Index | D = 1/2 SUM [blackpct - Whitepct] * 100, where blackpct is the proportion of the state’s Black population living in each block and Whitepct is the proportion of the state’s White population living in that block. Values are on a scale from 0-100 with 100 being the most spacially segregated by race. It represents the percentage of Black people who would have to move in order to achieve an equal distribution of White and Black people across all blocks within a state. | US Decennial Census, 2010 |
|  | Isolation Index | I = 100 - (SUM((blackpct)*(proportionblack)) * 100), where blackpct is the proportion of the state’s Black population living in each block and proportionblack is the proportion of people in that block who are Black. Values are on a scale from 0-100 with higher values representing higher levels of segregation. It can be interpreted as the probability a Black person does not share a block area with a White person or as the extent to which Black members of a block are exposed only to one another. | US Decennial Census, 2010 |
|  | Segregation Index | Average of Dissimilarity Index and Segregation Index |  |
| Incarceration | Incarceration Index | Ratio of Black incarceration rate to White incarceration rate for each state. Converted to 0-100 scale. | US Bureau of Justice Statistics, National Prisoner Statistics |
| Education | Education Index | Ratio of proportion of Black adults ages 25+ without a college degree to the proportion of White adults ages 25+ without a college degree for each state. Converted to 0-100 scale. | 2019 American Community Survey |
| Economic | Poverty Index | Ratio of proportion of Black people living under the poverty level to the proportion of White people living under the poverty level for each state. Converted to 0-100 scale. | 2019 American Community Survey |
|  | Income Index | Ratio of White median annual household income to Black median annual household income for each state. Converted to 0-100 scale. | 2019 American Community Survey |
|  | Rental Index | Ratio of proportion of Black people in rental housing to proportion of White people in rental housing for each state. Converted to 0-100 scale. | 2019 American Community Survey |
|  | Economic Index | Average of Poverty Index, Income Index, and Rental Index |  |
| Employment | Labor Force Participation Index | Ratio of proportion of Black people not participating in the labor force to proportion of White people not participating in the labor force for each state. Converted to 0-100 scale. | 2019 American Community Survey |
|  | Unemployment Index | Ratio of proportion of unemployment rate among Black people to the unemployment rate among White people for each state. Converted to 0-100 scale. | 2019 American Community Survey |
|  | Employment Index | Average of Labor Force Participation Index and Unemployment Index |  |
| Overall State Structural Racism Index | Racism Index | Average of Segregation Index, Incarceration Index, Education Index, Economic Index, and Employment Index |  |

**Appendix Table 3** Definitions, data sources, and methods for calculation of the potential mediating variables

| **Dimension** | **Measure** | **Description** | **Data source** |
| --- | --- | --- | --- |
| Disparities in potential exposure (operationalized as the ratio of the proportion of Black workers in each category to the proportion of White workers in each category) | Proportion of workers in jobs with likely exposure | Proportion of workers in the following job categories: Healthcare practitioners and technical occupations (29-0000); Healthcare support occupations (31-0000); and Protective service occupations (33-0000). | 2019 American Community Survey |
|  | Proportion of workers in essential jobs | Proportion of workers in the following job categories: Healthcare practitioners and technical occupations (29-0000); Healthcare support occupations (31-0000); Protective service occupations (33-0000); Food preparation and serving related occupations (35-0000); Building and grounds cleaning and maintenance occupations (37-0000); Personal care and service occupations (39-0000); Construction and extraction occupations (47-0000); Installation, maintenance and repair (49-0000); Production occupations (51-0000); and Transportation and material moving (53-0000). | 2019 American Community Survey |
| Disparities in weathering (operationalized as the ratio of proportion of the Black adults with co-morbidities affecting COVID-19 mortality risk to the proportion of White adults with that co-morbidity) | Hypertension | Proportion of adults with self-reported hypertension | 2019 Behavioral Risk Factor Surveillance System (BRFSS) surveys |
|  | Asthma | Proportion of adults with self-reported asthma | 2019 BRFSS |
|  | COPD | Proportion of adults with self-reported COPD | 2019 BRFSS |
|  | Obesity | Proportion of adults with self-reported obesity | 2019 BRFSS |
|  | Kidney disease | Proportion of adults with self-reported kidney disease | 2019 BRFSS |
|  | Cancer | Proportion of adults with self-reported cancer | 2019 BRFSS |
|  | Heart attack | Proportion of adults with self-reported heart attack | 2019 BRFSS |
|  | Angina | Proportion of adults with self-reported angina | 2019 BRFSS |
|  | Stroke | Proportion of adults with self-reported stroke | 2019 BRFSS |
|  | Any condition | Proportion of adults with 1+ of the above conditions | 2019 BRFSS |
|  | Two or more conditions | Proportion of adults with 2+ of the above conditions | 2019 BRFSS |
| Disparities in health care access (operationalized as the ratio of the proportion of the Black population without access/affordability to the proportion of the White population without access/affordability | Health insurance | Proportion of the population without health insurance | 2019 BRFSS |
|  | Health care affordability | Proportion of the population who reported not being able to afford medical care at some point in the past year | 2019 BRFSS |

**Appendix Table 4** Correlation matrix for main predictor variables

|  | State racism index | Black-White disparity in exposed jobs | Black-White disparity in essential jobs | Black-White disparity in any comorbidity | Black-White disparity in health insurance coverage | Black-White disparity in inability to afford health care |
| --- | --- | --- | --- | --- | --- | --- |
| State racism index | 1.00 |  |  |  |  |  |
| Black-White disparity in exposed jobs | 0.57 | 1.00 |  |  |  |  |
| Black-White disparity in essential jobs | 0.45 | 0.56 | 1.00 |  |  |  |
| Black-White disparity in any comorbidity | 0.25 | -0.11 | -0.22 | 1.00 |  |  |
| Black-White disparity in health insurance coverage | 0.73 | 0.64 | 0.58 | -0.19 | 1.00 |  |
| Black-White disparity in inability to afford health care | 0.34 | 0.39 | 0.32 | -0.05 | 0.52 | 1.00 |

**Appendix Table 5**  Overall state structural racism index and component indices

| **State** | **Racism index** | **Segregation index** | **Incarceration**  **index** | **Education index** | **Employment index** | **Economic index** | **Black/White death rate ratio** |
| --- | --- | --- | --- | --- | --- | --- | --- |
| Alabama | 35.0 | 72.8 | 1.4 | 33.5 | 36.6 | 30.8 | 1.9 |
| Arizona | 27.4 | 44.5 | 21.9 | 36.3 | 16.6 | 17.9 | 2.2 |
| Arkansas | 34.3 | 72.6 | 10.0 | 26.7 | 37.8 | 24.4 | 1.6 |
| California | 53.1 | 60.6 | 67.4 | 79.5 | 44.1 | 13.7 | 2.4 |
| Colorado | 52.7 | 53.0 | 42.3 | 99.7 | 43.7 | 24.8 | 2.7 |
| Connecticut | 60.3 | 63.5 | 69.7 | 100.0 | 30.8 | 37.8 | 2.7 |
| Florida | 35.2 | 66.4 | 16.2 | 56.1 | 19.1 | 18.0 | 3.0 |
| Georgia | 34.9 | 70.3 | 2.1 | 45.0 | 32.2 | 25.1 | 2.1 |
| Illinois | 61.0 | 77.9 | 50.7 | 69.1 | 60.0 | 47.1 | 2.5 |
| Indiana | 41.9 | 68.3 | 19.1 | 29.2 | 50.5 | 42.6 | 2.4 |
| Iowa | 52.4 | 52.7 | 59.5 | 53.1 | 38.6 | 58.1 | 2.5 |
| Kansas | 48.5 | 56.1 | 39.9 | 61.1 | 49.2 | 36.3 | 2.7 |
| Kentucky | 25.6 | 58.9 | 4.2 | 20.6 | 23.3 | 21.2 | 2.1 |
| Louisiana | 41.1 | 74.2 | 11.8 | 40.8 | 42.3 | 36.4 | 2.4 |
| Maryland | 46.7 | 73.2 | 26.9 | 70.4 | 38.5 | 24.4 | 2.6 |
| Massachusetts | 51.5 | 59.6 | 41.9 | 92.8 | 33.0 | 30.2 | 2.1 |
| Michigan | 51.9 | 75.1 | 40.2 | 46.4 | 50.7 | 47.3 | 4.5 |
| Minnesota | 62.6 | 57.5 | 70.8 | 67.1 | 35.9 | 81.4 | 3.2 |
| Mississippi | 39.4 | 74.8 | 0.0 | 34.0 | 45.2 | 42.8 | 2.1 |
| Missouri | 39.3 | 71.9 | 13.8 | 41.7 | 39.3 | 29.6 | 2.1 |
| Nebraska | 58.2 | 61.3 | 63.7 | 52.8 | 69.4 | 43.7 | 2.1 |
| Nevada | 31.1 | 45.8 | 12.4 | 44.1 | 31.8 | 21.6 | 2.6 |
| New Jersey | 66.3 | 70.8 | 100.0 | 90.1 | 38.8 | 31.8 | 2.3 |
| New York | 58.5 | 79.1 | 54.8 | 85.9 | 48.0 | 24.8 | 3.3 |
| North Carolina | 38.6 | 64.3 | 13.5 | 51.6 | 36.1 | 27.7 | 2.4 |
| Ohio | 48.6 | 68.6 | 30.6 | 45.3 | 53.3 | 45.0 | 1.9 |
| Oklahoma | 37.3 | 59.0 | 21.8 | 32.3 | 42.0 | 31.4 | 1.4 |
| Pennsylvania | 53.2 | 72.6 | 49.2 | 50.4 | 52.3 | 41.3 | 3.0 |
| Rhode Island | 50.6 | 54.3 | 60.3 | 59.2 | 43.9 | 35.1 | 1.8 |
| South Carolina | 43.4 | 66.9 | 13.4 | 62.1 | 36.1 | 38.4 | 2.3 |
| Tennessee | 33.7 | 71.1 | 8.4 | 30.1 | 33.0 | 25.7 | 2.1 |
| Texas | 34.7 | 65.0 | 8.5 | 54.7 | 31.0 | 14.2 | 2.0 |
| Virginia | 42.8 | 60.5 | 17.7 | 74.7 | 35.6 | 25.6 | 2.1 |
| Washington | 33.0 | 46.1 | 26.0 | 51.5 | 17.4 | 24.0 | 1.6 |
| Wisconsin | 72.3 | 74.2 | 96.0 | 63.9 | 67.0 | 60.3 | 2.9 |
